# Gender Differences in the Risk Factors for Endothelial Dysfunction in Chinese Hypertensive Patients: Homocysteine Is an Independent Risk Factor in Females

**DOI:** 10.1371/journal.pone.0118686

**Published:** 2015-02-18

**Authors:** Cheng Cao, Jianxin Hu, Yifei Dong, Rui Zhan, Ping Li, Hai Su, Qiang Peng, Tao Wu, Liang Lei, Xiao Huang, Qinghua Wu, Xiaoshu Cheng

**Affiliations:** 1 Department of Cardiovascular Medicine, Second Affiliated Hospital, Nanchang University, Nanchang City, Jiangxi Province, China; 2 Key Laboratory of Molecular Biology in Jiangxi Province, Nanchang City, Jiangxi Province, China; University of Utah School of Medicine, UNITED STATES

## Abstract

**Objectives:**

Endothelial dysfunction plays a key role in the pathogenesis of cardiovascular disease. However, the gender-related differences in risk factors for endothelial dysfunction are controversial. We investigated the gender differences in the risk factor profiles for endothelial dysfunction in Chinese hypertensive patients.

**Methods:**

Vascular endothelial functions in 213 hypertensive patients were measured by digital reactive hyperemia peripheral arterial tonometry (RH-PAT). Peripheral blood samples were collected, and the self-reported smoking and alcohol consumption status, age, body mass index, heart rate, blood pressure and drug administrations were recorded.

**Results:**

RH-PAT indexes were attenuated in both male and female hypertensive patients [1.60 (1.38-2.02) vs. 1.63 (1.44-1.98)]. Multivariate logistic regression analysis identified plasma creatinine (p < 0.001), total cholesterol (p = 0.001), homocysteine (p = 0.002) and smoking (p < 0.001) as the independent factors correlated with gender (male). Multivariate linear regression analysis further identified homocysteine as the factor that is significantly and independently correlated with the decrease in the RH-PAT indexes in female patients (odds ratio: -0.166, 95% confidence interval: -0.292 to -0.040, p = 0.01). However, none of these four factors were correlated with the RH-PAT indexes in male patients.

**Conclusions:**

There are gender-related differences in the risk factors for endothelial dysfunction in Chinese hypertensive patients. Homocysteine is an independent factor for endothelial dysfunction in female hypertensive patients.

## Introduction

The vascular endothelium plays a vital role in regulating the cardiovascular system and maintaining vascular health [1]. Endothelial dysfunction is an initiating event in the progression of cardiovascular diseases (CVD), and it is closely associated with the adverse clinical events of patients with established CVD and metabolic disorders [2, 3]. Over the past decade, gender differences in endothelial function have been observed and recognized for their use in gender-related cardiovascular disease [4]. Gender differences in the burden of CVD influence the relationship between endothelial dysfunction and the adverse outcomes of CVD. They also provide evidence for the importance of gender-based CVD prevention [5].

Endothelial dysfunction occurs in response to vascular risk factors. The traditional risk factors for CVD are overall similar in men and women [6]. However, there are gender differences in the prevalence of traditional risk factors. The National Health and Nutrition Examination Survey (NHANES) showed that the prevalence of high blood pressure is higher in women > 65 years of age [7]. Diabetes mellitus is more prevalent among women than men ≥ 20 years of age (8.3% vs. 7.2%) [8]. The age-adjusted prevalence of metabolic syndrome is highest among Mexican American women (40.6%), which is ≈ 22% higher than in Mexican American men [9]. The prevalence of total cholesterol ≥ 240 mg/dl for those ≥ 20 years of age was 16.2% among women and 13.5% among men [8]. Novel biomarkers representing new risk factors, such as homocysteine (Hcy), correlate with endothelial dysfunction in humans [10]. The impact of these new risk factors on vascular disease seem to vary by gender, race and ethnicity [11, 12]. Therefore, the presence of gender differences in the risk factor profiles might be one of the most important factors influencing the gender basis of CVD prevention. However, the influence of risk factor profiles on endothelial function, especially gender-related differences in endothelial dysfunction, remains to be identified. The aims of this study were to investigate the relationship between vascular risk factors and endothelial function in male and female Chinese hypertensive patients.

## Methods

### Study subjects

We evaluated 229 consecutive hypertensive patients who underwent peripheral endothelial function measurements at the Second Affiliated Hospital of Nanchang University from June 2013 to July 2014. Primary hypertension was defined as being treated with anti-hypertensive drugs or having a systolic blood pressure (SBP) ≥ 140 mmHg and/or diastolic blood pressure (DBP) ≥ 90 mmHg. Hyperhomocysteinemia was defined as a total plasma homocysteine concentration ≥ 10 μmol/l. Exclusion criteria were secondary hypertension; previous diagnosis of diabetes mellitus; Parkinson’s disease; acute myocardial infarction; pulseless disease; severe systemic diseases, including systemic lupus erythematosus (SLE), or severe liver or renal disease; or any other situation that makes them unsuitable for participating in this study. We excluded 16 patients who met the exclusion criteria. Our final study sample included 113 male hypertensive patients and 100 female hypertensive patients.

The study was conducted according to the guidelines of the Declaration of Helsinki and was approved by the Medical Research Ethics Committee of Second Affiliated Hospital of Nanchang University, and we obtained a signed informed consent from each patient before participation.

### Clinical data collection

A standardized medical history and accurate physical examination were obtained from all subjects. The self-reported smoking and alcohol consumption statuses were recorded. Height and weight were measured in a standing position without shoes. The body mass index (BMI) was calculated as the weight (in kilograms) divided by the height (in meters squared). After a 12-h fast (no alcohol), a peripheral blood sample was collected. The levels of creatinine, uric acid, fasting plasma glucose, total cholesterol (TC), triglyceride (TG), high-density lipoprotein (HDL) cholesterol, low-density lipoprotein (LDL) cholesterol and Hcy were measured with a standard assays for all subjects. The estimated glomerular filtration rate (eGFR) was calculated according to the modified glomerular filtration rate estimating equation for Chinese patients with chronic kidney disease [13]. The serum creatinine concentrations were measured by an enzymatic method using test kits (Biote Co., Ltd., Nanchang, China), and the serum uric acid concentrations were assayed by a direct enzyme method (Medical Co., Ltd., Ningbo, China). The fasting plasma glucose level was measured using the hexokinase/glucose-6-phosphate dehydrogenase method (Biote Co., Ltd., Nanchang, China). The TC levels were determined with the enzymatic colorimetric method (Medical Co., Ltd., Ningbo, China), TG levels were evaluated by the GPO-POD method (Beckman Coulter, Suzhou, China), HDL cholesterol and LDL cholesterol were measured by direct homogeneous assay methods using detergents (Medical Co., Ltd., Ningbo, China), and plasma Hcy was determined by enzymatic methods using test kits (AUSA Co., Ltd., Shenzhen, China). All of the biochemical variables were measured using an auto-analyzer (OLYMPUS AU-2700) at the central laboratory of the Second Affiliated Hospital of Nanchang University.

### Measurement of endothelial function

Peripheral endothelial function was assessed using an Endo-PAT2000 device (Itamar Medical, Caesarea, Israel), as described previously [14]. All tests were performed in the morning in a quiet, air-conditioned room with a temperature of 22–24°C. Patients were asked to fast for 12 hrs and instructed to refrain from caffeine, smoking, alcohol and strenuous exercise for at least 12 hrs before the examination.

Before measurement, patients were asked to rest for 10 min. The peripheral blood pressure was measured three times in the right brachial artery (Omron, HEM-7112); the measurements were performed at a 2-min interval, and the average SBP and DBP measurements were used for analysis and further calculation. Briefly, one PAT finger probe was placed on the index finger of the hand undergoing hyperemia testing (right hand), and a second PAT probe was placed on the contralateral index finger (left hand). A blood pressure cuff was placed on one upper arm (right hand) at whichever occlusion pressure would be higher, 220 or 60 mmHg plus systolic blood pressure. This arm was the study arm, whereas the other arm served as a control arm. The signals were measured from each fingertip at the basal state, for 5 minutes; then, the arterial flow was interrupted for 5 minutes. The pulse amplitude was recorded for 10 minutes electronically in both fingers during cuff occlusion and following cuff deflation. Typical tracings are represented in S1 Fig. The data were digitized and computed automatically with Endo-PAT2000 software; the digital reactive hyperemia peripheral arterial tonometry (RH-PAT) index, representing the endothelial function, was defined as the ratio of the mean post-deflation signal (in the 90 to 120-second post-deflation interval) to the baseline signal in the hyperemic finger and was normalized by the same ratio in the contra-lateral finger and was multiplied by a baseline correction factor, as calculated by the Endo-PAT2000 software. An RH-PAT index ≤1.67 was considered abnormal.

### Statistical analysis

Normally distributed results were expressed as the mean ± standard deviation (SD) values. The values of the RH-PAT index, age, BMI, plasma total Hcy, uric acid, creatinine, eGFR, TC, TG, HDL cholesterol, LDL cholesterol, DBP, and fasting blood glucose were not normally distributed and were expressed as the median value (interquartile range) and were log transformed before Pearson’s correlation and regression analyses. Categorical values were presented as the numbers (percentage) and compared by the chi-square test. Comparisons of continuous variables were analyzed by the independent-samples T-test or Mann-Whitney U-test, as appropriate. To determine the effect of a gender difference on the risk factors, multivariate logistic regression analysis with a Forward-Wald variable selection method was performed. Linear relationships between the RH-PAT index and risk factors were first analyzed by univariate analysis, which was followed by stepwise multivariate linear regression to assess the clinical marker correlated with the RH-PAT index in male and female hypertensive patients, respectively. Two multivariate linear regression analyses for the log RH-PAT were developed; model 1 included the variables that were statistically significant in the univariate analyses and model 2 included the factors that were independently correlated with gender differences. Pearson’s correlation was used for simple linear analysis between the RH-PAT index and plasma Hcy. A 2-sided p-value of < 0.05 was considered significant. All statistical analyses were performed with SPSS software for Windows, version 16.0 (SPSS, Chicago, Illinois).

## Results

### Clinical characteristics of the study sample

Table 1 details the clinical characteristics of all of the study subjects. There were no significant differences in the age, BMI, eGFR, fasting blood glucose, TG, LDL cholesterol, heart rate or blood pressure between male and female patients. The RH-PAT indexes were attenuated in both the male and female hypertensive patients. More of the males were smokers and alcohol consumers. Compared with males, female patients had higher TC (p = 0.001) and HDL cholesterol (p < 0.001) levels, and they had lower levels of uric acid (p < 0.001), creatinine (p < 0.001), and homocysteine (p < 0.001) as well as a lower ratio of aspirin administration (p = 0.03).

**Table 1:** Clinical Characteristics of Male and Female Hypertensive Patients.

	Hypertensive Patients (n = 213)		
	Male Patients	Female Patients	p Value
	(n = 113)	(n = 100)	
Age (yrs)	63.00 (54.00, 69.00)	63.50 (57.00, 72.00)	0.224
Body mass index (kg/m^2^)	24.49 (21.45, 26.61)	24.49 (22.14, 27.34)	0.694
Uric acid (mg/dl)	6.20 (5.27, 7.03)	5.12 (4.14, 6.08)	< 0.001
Creatinine (mg/dl)	0.95 (0.82, 1.13)	0.71 (0.63, 0.83)	< 0.001
eGFR (ml/min/1.73 m2)	105.63 (86.30, 126.75)	108.76 (90.99, 126.70)	0.262
Fasting blood glucose (mg/dl)	92.26 (85.78, 100.55)	94.79 (87.31, 106.59)	0.074
Total cholesterol (mg/dl)	159.71 (143.08, 196.44)	185.81 (157.58, 215.78)	0.001
Triglyceride (mg/dl)	109.79 (81.46, 161.15)	124.40 (87.66, 176.20)	0.131
HDL cholesterol (mg/dl)	34.42 (30.55, 42.54)	40.80 (34.03, 46.60)	< 0.001
LDL cholesterol (mg/dl)	103.63 (81.21, 129.54)	115.24 (90.29, 139.02)	0.057
Homocysteine (umol/L)	15.67 (11.96, 19.23)	10.68 (8.39, 13.42)	< 0.001
RH-PAT index	1.60 (1.38, 2.02)	1.63 (1.44, 1.98)	0.556
Heart rate (beats/min)	70.95 ± 12.15	71.84 ± 11.14	0.587
SBP (mmHg)	139.49 ± 19.00	138.70 ± 20.19	0.769
DBP (mmHg)	80.00 (71.00, 85.00)	75.84 (68.00, 83.67)	0.166
Current smoking [n (%)]	52 (46.0%)	4 (4.0%)	< 0.001
Current alcohol use [n (%)]	37 (32.7%)	3 (3.0%)	< 0.001
Medications			
Beta-blockers [n (%)]	38 (33.6%)	23 (23.0%)	0.087
ACIEs or ARBs [n (%)]	83 (73.5%)	79 (79.0%)	0.344
CCBs [n (%)]	89 (78.8%)	84 (84.0%)	0.329
Statins [n (%)]	67 (59.3%)	50 (50.0%)	0.174
Aspirin [n (%)]	49 (43.4%)	29 (29.0%)	0.030
Diuretics [n (%)]	30 (26.5%)	19 (19.0%)	0.191

Abbreviations: eGFR, estimated glomerular filtration rate; LDL, low-density lipoprotein; HDL, high-density lipoprotein; RH-PAT, reactive hyperemia peripheral arterial tonometry; SBP, systolic blood pressure; DBP, diastolic blood pressure; ACEI, angiotensin-converting enzyme inhibitor; ARB, angiotensin II receptor blocker; CCB, calcium channel blocker.

Continuous variables, the mean ± SD or median (interquartile range); Dichotomous variables, NO. (%).

### The effect of gender on the clinical factors in hypertensive patients

Table 2 shows the result of logistic regression analysis for gender (male) in all hypertensive patients. Univariate logistic regression analysis showed that smoking, alcohol consumption, the ratio of aspirin administration and the levels of uric acid, creatinine, log(TC), log(HDL) and log(Hcy) correlated with male hypertensive patients. Multivariate logistic regression analysis further identified that smoking (odds ratio [OR]: 16.626, 95% confidence interval [CI]: 5.273 to 52.428, p < 0.001) and the levels of creatinine (OR: 25.264, 95% CI: 4.944 to 129.104, p < 0.001), log(TC) (OR: 0.001, 95% CI: 0.000 to 0.069, p = 0.001) and log(Hcy) (OR: 34.107, 95% CI: 3.701 to 314.325, p = 0.002) were independently correlated with gender (male) in all of the hypertensive patients.

**Table 2:** Univariable and Forward-Wald Logistic Regression Analysis of the Clinical Factors for Gender (Male) among Hypertensive Patients.

Variable	Univariate		Multivariate	
	OR (95% CI)	p Value	OR (95% CI)	p Value
Log Age	0.094 (0.005, 1.751)	NS (0.113)	Not selected	
Log Body mass index	0.455 (0.012, 16.997)	NS (0.670)	Not selected	
Uric acid	1.610 (1.311, 1.977)	< 0.001	**…**	
Creatinine	73.057 (15.735, 339.201)	< 0.001	25.264 (4.944, 129.104)	< 0.001
Log eGFR	0.401 (0.061, 2.650)	NS (0.343)	Not selected	
Log FBG	0.044 (0.001, 1.552)	NS (0.086)	Not selected	
Log TC	0.006 (0.000, 0.112)	0.001	0.001 (0.000, 0.069)	0.001
Log TG	0.400 (0.112, 1.426)	NS (0.158)	Not selected	
Log LDL	0.143 (0.020, 1.016)	NS (0.052)	Not selected	
Log HDL	0.009 (0.001, 0.119)	< 0.001	**…**	
Log Hcy	224.200 (31.966, 1572.461)	< 0.001	34.107 (3.701, 314.325)	0.002
Log RH-PAT	0.179 (0.018, 1.794)	NS (0.143)	Not selected	
Heart rate	0.993 (0.971, 1.017)	NS (0.576)	Not selected	
SBP	1.002 (0.988, 1.016)	NS (0.768)	Not selected	
Log DBP	18.659 (0.415, 838.839)	NS (0.132)	Not selected	
Current smoking	20.459 (7.043, 59.432)	< 0.001	16.626 (5.273, 52.428)	< 0.001
Current alcohol use	15.741 (4.674, 53.014)	< 0.001	**…**	
Medications				
Beta-blockers	1.696 (0.924, 3.114)	NS (0.088)	Not selected	
ACEIs or ARBs	0.735 (0.389, 1.391)	NS (0.344)	Not selected	
CCBs	0.706 (0.351, 1.421)	NS (0.330)	Not selected	
Statins	1.457 (0.847, 2.506)	NS (0.174)	Not selected	
Aspirin	1.874 (1.060, 3.315)	0.031	**…**	
Diuretics	1.541 (0.804, 2.955)	NS (0.193)	Not selected	

Abbreviations: eGFR, estimated glomerular filtration rate; FBG, fasting blood glucose; TC, total cholesterol; TG, triglycerides; LDL, low-density lipoprotein; HDL, high-density lipoprotein; Hcy, homocysteine; RH-PAT, reactive hyperemia peripheral arterial tonometry; SBP, systolic blood pressure; DBP, diastolic blood pressure; ACEI, angiotensin-converting enzyme inhibitor; ARB, angiotensin II receptor blocker; CCB, calcium channel blocker; OR, odds ratio; CI, confidence interval.

Dashes indicate that the variable did not enter the Forward-Wald model; NS, not significant.

### Correlation of the clinical factors with endothelial function in male and female hypertensive patients

Table 3 details the univariate and multivariate linear regression analysis for clinical factors and the RH-PAT index in female hypertensive patients. Univariate linear regression analysis showed that the levels of log(HDL) and log(Hcy) correlated with the log(RH-PAT index) in female hypertensive patients. The multivariate linear regression model 1 further identified that log(HDL) (beta = 0.250, 95% CI: 0.049 to 0.450, p = 0.015) and log(Hcy) (beta = -0.132, 95% CI: -0.257 to-0.006, p = 0.041) were independently and significantly correlated with the log(RH-PAT index) in female hypertensive patients. Model 2 included creatinine, log(TC), log(Hcy) and smoking, which were identified as being independently correlated to the gender difference in all hypertensive patients. Only the log(Hcy) level was independently and significantly correlated with the log(RH-PAT index) in female hypertensive patients (beta = -0.166, 95% CI: -0.292 to -0.040, p = 0.01).

**Table 3:** Univariate and Stepwise Multivariate Linear Regression Analyses for the Log RH-PAT in Female Patients.

	Univariate Analysis		Multivariate Analysis		Multivariate Analysis	
			Model 1, r = 0.347		Model 2, r = 0.255	
Variable	β (95% CI)	p Value	β (95% CI)	p Value	β (95% CI)	p Value
Log Age	0.219 (-0.049, 0.487)	NS (0.108)	Not selected		Not selected	
Log Body mass index	-0.102 (-0.396, 0.193)	NS (0.495)	Not selected		Not selected	
Uric acid	-0.006 (-0.021, 0.009)	NS (0.438)	Not selected		Not selected	
Creatinine	-0.025 (-0.107, 0.058)	NS (0.555)	Not selected		**…**	
Log eGFR	-0.018 (-0.165, 0.129)	NS (0.810)	Not selected		Not selected	
Log FBG	-0.195 (-0.465, 0.074)	NS (0.153)	Not selected		Not selected	
Log TC	0.115 (-0.120, 0.349)	NS (0.335)	Not selected		**…**	
Log TG	-0.084 (-0.184, 0.017)	NS (0.102)	Not selected		Not selected	
Log LDL	0.024 (-0.138, 0.186)	NS (0.769)	Not selected		Not selected	
Log HDL	0.296 (0.097, 0.495)	0.004	0.250 (0.049, 0.450)	0.015	Not selected	
Log Hcy	-0.166 (-0.292, -0.040)	0.010	-0.132 (-0.257, -0.006)	0.041	-0.166 (-0.292, -0.040)	0.010
Heart rate	< 0.001 (-0.003, 0.001)	NS (0.423)	Not selected		Not selected	
SBP	0.001 (0.000, 0.002)	NS (0.068)	Not selected		Not selected	
Log DBP	-0.235 (-0.535, 0.065)	NS (0.123)	Not selected		Not selected	
Current smoking	0.033 (-0.081, 0.146)	NS (0.567)	Not selected		**…**	
Current alcohol use	-0.114 (-0.243, 0.015)	NS (0.082)	Not selected		Not selected	
Medications						
Beta-blockers	-0.012 (-0.065, 0.041)	NS (0.654)	Not selected		Not selected	
ACEIs or ARBs	0.011 (-0.043, 0.066)	NS (0.685)	Not selected		Not selected	
CCBs	0.016 (-0.045, 0.076)	NS (0.609)	Not selected		Not selected	
Statins	0.025 (-0019, 0.069)	NS (0.269)	Not selected		Not selected	
Aspirin	-0.024 (-0.073, 0.024)	NS (0.324)	Not selected		Not selected	
Diuretics	-0.049 (-0.105, 0.007)	NS (0.084)	Not selected		Not selected	

Abbreviations: eGFR, estimated glomerular filtration rate; FBG, fasting blood glucose; TC, total cholesterol; TG, triglycerides; LDL, low-density lipoprotein; HDL, high-density lipoprotein; Hcy, homocysteine; RH-PAT, reactive hyperemia peripheral arterial tonometry; SBP, systolic blood pressure; DBP, diastolic blood pressure; ACEI, angiotensin-converting enzyme inhibitor; ARB, angiotensin II receptor blocker; CCB, calcium channel blocker; β, regression coefficient; CI, confidence interval.

Dashes indicate that the variable did not enter the stepwise multivariate linear regression model; NS, not significant.

Table 4 details the univariate and multivariate linear regression analysis for the clinical factors and RH-PAT index in male hypertensive patients. Unlike for female patients, none of the four identified risk factors [creatinine, log(TC), log(Hcy) and smoking] were correlated with the log(RH-PAT index) in male hypertensive patients.

**Table 4:** Univariate and Stepwise Multivariate Linear Regression Analyses for the Log RH-PAT in Male Patients.

	Univariate Analysis		Multivariate Analysis		Multivariate Analysis	
			Model 1, r = 0.231		Model 2	
Variable	β (95% CI)	p Value	β (95% CI)	p Value	β (95% CI)	p Value
Log Age	0.189 (-0.023, 0.400)	NS (0.080)	Not selected		Not selected	
Log Body mass index	0.062 (-0.254, 0.379)	NS (0.696)	Not selected		Not selected	
Uric acid	-0.014 (-0.029, 0.001)	NS (0.072)	Not selected		Not selected	
Creatinine	-0.049 (-0.121, 0.023)	NS (0.182)	Not selected		**…**	
Log eGFR	0.067 (-0.102, 0.236)	NS (0.433)	Not selected		Not selected	
Log FBG	0.185 (-0.121, 0.492)	NS (0.233)	Not selected		Not selected	
Log TC	0.057 (-0.173, 0.287)	NS (0.626)	Not selected		**…**	
Log TG	< 0.001 (-0.111, 0.110)	NS (0.994)	Not selected		Not selected	
Log LDL	-0.002 (-0.166, 0.162)	NS (0.980)	Not selected		Not selected	
Log HDL	0.175 (-0.023, 0.373)	NS (0.083)	Not selected		Not selected	
Log Hcy	-0.050 (-0.169, 0.069)	NS (0.409)	Not selected		**…**	
Heart rate	< 0.001 (-0.002, 0.002)	NS (0.886)	Not selected		Not selected	
SBP	0.001 (0.000, 0.002)	NS (0.319)	Not selected		Not selected	
Log DBP	-0.133 (-0.463, 0.198)	NS (0.428)	Not selected		Not selected	
Current smoking	0.003 (-0.044, 0.049)	NS (0.910)	Not selected		**…**	
Current alcohol use	-0.017 (-0.067, 0.032)	NS (0.486)	Not selected		Not selected	
Medications						
Beta-blockers	-0.060 (-0.108, -0.013)	0.014	-0.060 (-0.108, -0.013)	0.014	Not selected	
ACEIs or ARBs	-0.019 (-0.071, 0.033)	NS (0.475)	Not selected		Not selected	
CCBs	0.059 (0.004, 0115)	0.037	**…**		Not selected	
Statins	0.007 (-0040, 0.055)	NS (0.754)	Not selected		Not selected	
Aspirin	0.004 (-0.043, 0.051)	NS (0.876)	Not selected		Not selected	
Diuretics	-0.043 (-0.095, 0.009)	NS (0.107)	Not selected		Not selected	

Abbreviations: eGFR, estimated glomerular filtration rate; FBG, fasting blood glucose; TC, total cholesterol; TG, triglycerides; LDL, low-density lipoprotein; HDL, high-density lipoprotein; Hcy, homocysteine; RH-PAT, reactive hyperemia peripheral arterial tonometry; SBP, systolic blood pressure; DBP, diastolic blood pressure; ACEI, angiotensin-converting enzyme inhibitor; ARB, angiotensin II receptor blocker; CCB, calcium channel blocker; β, regression coefficient; CI, confidence interval.

Dashes indicate that the variable did not enter the stepwise multivariate linear regression model; NS, not significant.

**Fig 1:**
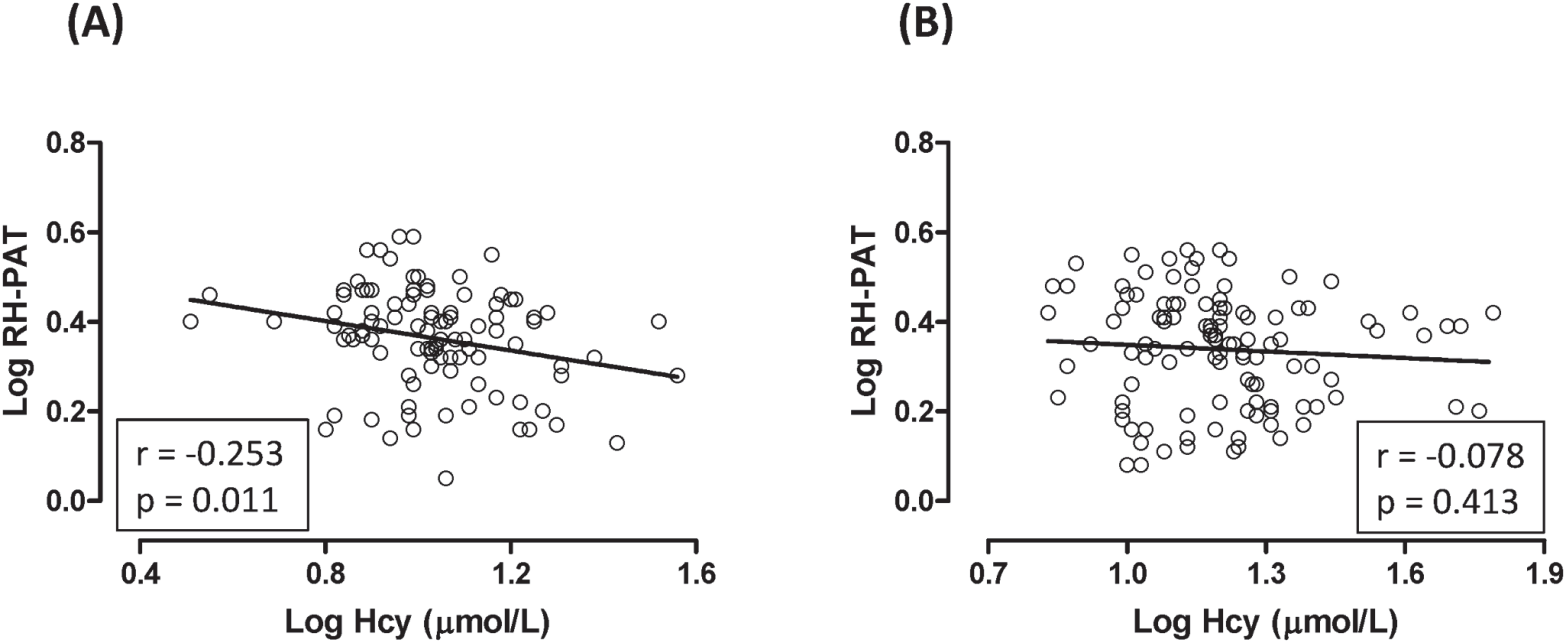
Correlation of the Log RH-PAT Index with the Log Hcy in female (A) and male (B) hypertensive patients. Fig. 1 shows the simple linear analysis results of the correlation between the log(Hcy) and log(RH-PAT index) in female (Fig. 1A) (r = -0.253, p = 0.011) and male (Fig. 1B) (r = -0.078, p = 0.413) hypertensive patients, respectively.

Fig. 1 shows the simple linear analysis result of the correlation between the log(Hcy) and log(RH-PAT index) in female (Fig. 1A) (r = -0.253, p = 0.011) and male (Fig. 1B) (r = -0.078, p = 0.413) hypertensive patients.

## Discussion

This study reports the first vascular risk factor profile for endothelial dysfunction in male and female Chinese hypertensive patients. There was a gender difference in the association between risk factors and endothelial dysfunction. More importantly, we identified that an elevated Hcy level, a non-traditional risk factor, was significantly correlated with endothelial dysfunction in female patients.

Gender-related differences in endothelial dysfunction are supposed to be related to age and hormonal status [15]. Compared with men, endothelial dysfunction occurs late in women. Women have better peripheral endothelial function than men until approximately age 70 [16, 17]. In our study, RH-PAT indexes were attenuated in both male and female hypertensive patients and they have a tendency to be lower in males. In addition to age, there is also endothelial dysfunction in response to other vascular risk factors. However, a recent study investigating the gender differences associated with changes in brachial artery flow-mediated dilatation by the Framingham Risk Score in 1038 Chinese patients showed that there is a statistically significant difference in flow-mediated dilatation between women and men with the same Framingham risk level [18]. This result indicates that the traditional risk factor profiles are not sufficient to explain the gender difference in endothelial dysfunction. In our study, the traditional risk factors, including age, BMI, smoking, alcohol consumption, TC, TG, LDL cholesterol, systolic blood pressure and diastolic blood pressure, were not correlated with endothelial dysfunction in both male and female patients.

Consistent with the previous population-based studies [19, 20], the plasma total Hcy level, a novel identified risk factor for endothelial dysfunction and CVD [10], was significantly higher in males than in females. Higher remethylation rates and higher transmethylation in females were suggested as contributing to this phenomenon [21]. However, it is worth noting that the plasma total Hcy level was significantly correlated with endothelial dysfunction in only female hypertensive patients.

The gender difference of the relationship between the plasma total Hcy and endothelial dysfunction in hypertensive patients may be clinically important. Over the past decade, researchers have made substantial efforts to improve our understanding of the gender differences in CVD and other vascular diseases, such as stroke. With respect to the vital role of endothelial dysfunction in vascular diseases, gender-related differences in endothelial dysfunction could be a potential tool in gender-related cardiovascular diseases [4]. Gender differences in the burden of vascular disease have been demonstrated in multiple epidemiologic and cohort studies [8, 22, 23]. According to the report by the American Heart Association in 2011, the absolute numbers of women living with and dying of CVD and stroke exceed those of men [8]. In 2007, women accounted for 60.6% of US stroke deaths [8]. However, the gender difference in the relationship between vascular events and the Hcy level in hypertensive patients is still controversial. In 2004, a Northern Manhattan study (NOMAS), which was a population-based study in which 73.3% of the participants were hypertensive patients, demonstrated that there were no significant differences in the association between the plasma total Hcy level and vascular outcomes by gender [24]. In contrast, a recent community-based cross-sectional study conducted in Chinese hypertensive patients showed a higher odds ratio of ischemic stroke for the total plasma Hcy level ≥ 30 (vs. ≤ 15) μmol/L in women compared with male hypertensive patients (4.41 vs. 2.84) [25]. The significant and inverse correlation between the total plasma Hcy level and endothelial dysfunction in female Chinese hypertensive patients was identified in our study as one potential cause accounting for the different burden of stroke in male and female Chinese hypertensive patients.

As a novel marker and new vascular risk factor, the total plasma Hcy plasma has been demonstrated to be independently correlated with vascular disease [24, 26]. Notably, the relationship between the Hcy level and vascular disease, especially stroke, varies by gender, race and ethnicity [12]. Comparing the risk factor profiles of vascular diseases with US, a significantly increased plasma total Hcy level in the Chinese population has been acknowledged [27]. It might be one of the key causes accounting for the continuing increase of stroke incidence in Chinese individuals; therefore, it is supposed to be clinically important in China.

Endothelial function is impaired across the stages of the menopause transition in healthy women. A decline in endothelial function begins during the early stages of menopause (perimenopause) and worsens with the loss of ovarian function and prolonged estrogen deficiency [28]. Long-term hormone replacement therapy results in lower total Hcy concentrations in postmenopausal women [29]. Therefore, it could be supposed that the menopausal status may influence the correlation between endothelial function and Hcy concentration. Although we did not record the menopausal status in the study, the median age of the female patients enrolled in the study were post-menopausal. This fact should be taken into consideration that it would potentially strength the correlation between endothelial dysfunction and Hcy level in this study.

Low HDL cholesterol, one of the conventional risk factors for CVD, has been well established as a risk factor for endothelial dysfunction[30]. Traditionally, low HDL cholesterol has been considered to be a gender-neutral cardiometabolic risk factor [31]. To the best of our knowledge, no studies have investigated the gender-specific impact of HDL cholesterol on endothelial function. In our study, HDL cholesterol was identified as an independent factor that correlates with the RH-PAT index in only female hypertensive patients (Tables 3 and 4). However, at the same time, it was not identified as a factor that independently correlates with gender differences in all patients (Table 2). The relatively small number of patients in the study should be considered to underestimate the correlation of HDL cholesterol and gender difference. However, as far as the potential protective effect and the debated role of HDL cholesterol in vascular diseases are concerned [32], the gender difference based effect of HDL cholesterol on endothelial dysfunction, identified in our study, merits further investigation in future studies.

The present study has certain limitations. There is a complete reversal of cardiovascular risk in women pre- vs post-menopause as well as in the years post-menopause [8,33–36]. However, the menopausal status was not determined in this study, which is critical, especially because there seem to be significant discrepancies between the data, populations and published literature [37–39]. Therefore, menopausal status should be noted and clarified in future studies. In addition, this study investigated only a relatively small number of patients from a single center and was limited to Chinese patients with hypertension. However, this should result in an underestimation of the findings, emphasizing the need for further multicenter studies in a larger population and different races to confirm the present results.

In conclusion, the present study demonstrates that there are gender-related differences in the risk factors for endothelial dysfunction in Chinese hypertensive patients. An elevated total plasma Hcy level and reduced HDL cholesterol level were identified as risk factors that independently correlate with endothelial dysfunction in female hypertensive patients.

## Supporting Information

S1 Fig Illustration of typical tracings in female hypertensive patients. **A** and **B**, pulse amplitude recordings with PAT at baseline, during arterial occlusion (with a brachial cuff), and during reactive hyperemia (after cuff deflation) in the ischemic arm. **C**, PAT recording from the contralateral finger not undergoing reactive hyperemia testing. **A**, female hypertensive patients with normal blood homocysteine showing a steady-state PAT signal (baseline) and complete disappearance of the signal during cuff inflation (occlusion), which is followed by an increased PAT signal during recovery (hyperemia) (RH-PAT = 2.60). **B**, female hypertensive patients with hyperhomocysteinemia showing a blunted finger PAT response during reactive hyperemia (RH-PAT = 0.71). **C**, PAT recording from the contralateral finger in the same patient with hypertension and hyperhomocysteinemia.
(TIFF)
