# Supplementary figures and images for: Gender Differences in the Risk Factors for Endothelial Dysfunction in Chinese Hypertensive Patients: Homocysteine Is an Independent Risk Factor in Females
Source: PLoS One. 2015 Feb 18;10(2):e0118686. doi: 10.1371/journal.pone.0118686 (PMC4334966; doi:10.1371/journal.pone.0118686)

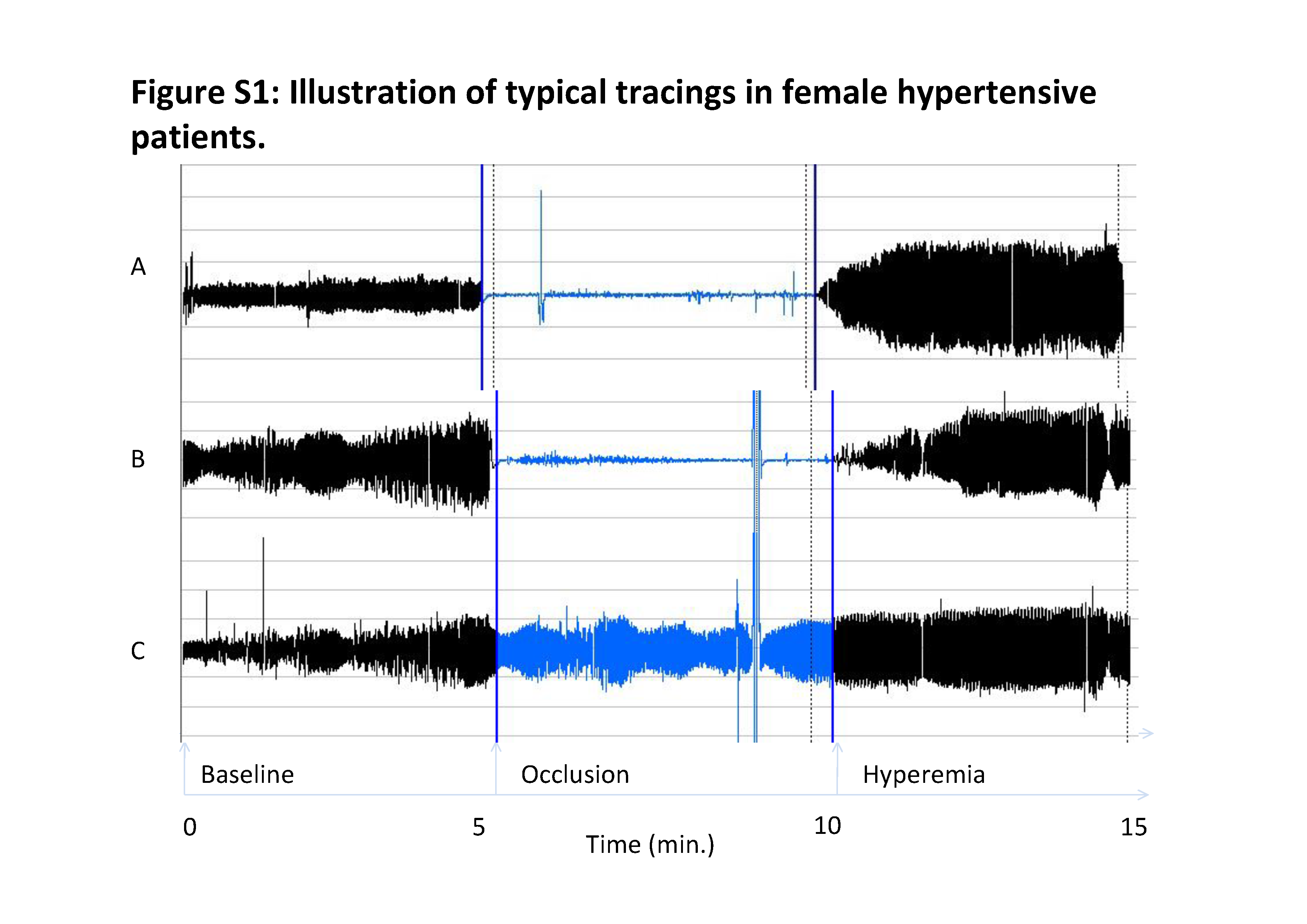

Supplement: S1 Fig — A and B, pulse amplitude recordings with PAT at baseline, during arterial occlusion (with a brachial cuff), and during reactive hyperemia (after cuff deflation) in the ischemic arm. C, PAT recording from the contralateral finger not undergoing reactive hyperemia testing. A, female hypertensive patients with normal blood homocysteine showing a steady-state PAT signal (baseline) and complete disappearance of the signal during cuff inflation (occlusion), which is followed by an increased PAT signal during recovery (hyperemia) (RH-PAT = 2.60). B, female hypertensive patients with hyperhomocysteinemia showing a blunted finger PAT response during reactive hyperemia (RH-PAT = 0.71). C, PAT recording from the contralateral finger in the same patient with hypertension and hyperhomocysteinemia. (TIFF) [file pone.0118686.s001.tiff]
